# Crystal structure of di-μ-aqua-μ-(pyrazine *N*,*N*′-dioxide)-κ^2^ *O*:*O*-bis­(di­aqua­sodium) tetra­phenyl­borate dihydrate pyrazine *N*,*N*′-dioxide monosolvate

**DOI:** 10.1107/S205698901502071X

**Published:** 2015-11-07

**Authors:** Elaine P. Boron, Kelsey K. Carter, Jacqueline M. Knaust

**Affiliations:** a Chemistry Department, 520 North Main St., Meadville, PA 16335, USA; b Department of Chemistry Mathematics and Physics, Clarion University, 840 Wood Street, Clarion, PA 16214, USA

**Keywords:** crystal structure, sodium coordination compound, pyrazine *N,N′*-dioxide (pzdo), C—H⋯O inter­actions, O—H⋯O inter­actions, C—H⋯π inter­actions, O—H⋯π inter­actions

## Abstract

The search for novel lanthanide coordination networks using pyrazine *N,N′*-dioxide (pzdo, C_4_H_4_N_2_O_2_) as a structure-directing unit, led to the synthesis and the structure determination of the title compound, [Na_2_(C_4_H_4_N_2_O_2_)(H_2_O)_6_][B(C_6_H_5_)_4_]_2_·C_4_H_4_N_2_O_2_·2H_2_O. The crystal structure is comprised of discrete [{Na(H_2_O)_2_}_2_(μ-H_2_O)_2_(μ-pzdo)]^2+^ cations and tetra­phenyl­borate anions, as well as pzdo and H_2_O solvent mol­ecules. The dinuclear cation is located about a twofold rotation axis, and the symmetry-related Na^I^ atoms display a distorted square-pyramidal coordination sphere defined by two O atoms of terminal water ligands, two O atoms of bridging water ligands and one O atom of a bridging pzdo ligand. In the crystal, O—H⋯O hydrogen bonds link the dinuclear cation and solvent pzdo mol­ecules (point-group symmetry -1) into rectangular grid-like layers parallel to the *bc* plane. Additional C—H⋯O, O—H⋯O, C—H⋯π and O—H⋯π inter­actions link the anion and solvent water mol­ecules to the layers. The layers are further linked into a three-dimensional network through a combination of C—H⋯π and O—H⋯π hydrogen bonds involving the tetra­phenyl­borate anion.

## Abstract 2

The crystal structure of the title compound contains discrete [{Na(H_2_O)_2_}_2_(μ-H_2_O)_2_(μ-pzdo)]^2+^ (pzdo is pyrazine *N*,*N*′-dioxide) cations, tetra­phenyl­borate anions and uncoordinating water and pzdo mol­ecules, held together by various hydrogen-bonding and C—H⋯π and O—H⋯π inter­actions.

## Chemical context   

The use of aromatic *N,N′*-dioxide ligands such as pyrazine *N,N*′-dioxide (pzdo) and 4,4′-pyridine-*N,N*′-dioxide (bpydo) in the synthesis of transition metal and lanthanide metal compounds with coordination networks has been of recent inter­est (Hill *et al.*, 2005*b* ▸; Ma *et al.*, 2001 ▸; Mantero *et al.*, 2006 ▸; Sun *et al.*, 2004 ▸). The coordination modes and hydrogen-bonding modes of *N,N′*-dioxide ligands are flexible (Ma *et al.*, 2001 ▸; Mantero *et al.*, 2006 ▸). Structure prediction with these ligands can be difficult, in part due to their flexible bonding, but also due to the influences of the anion and solvent (Hill *et al.*, 2005*a* ▸; Mantero *et al.*, 2006 ▸).
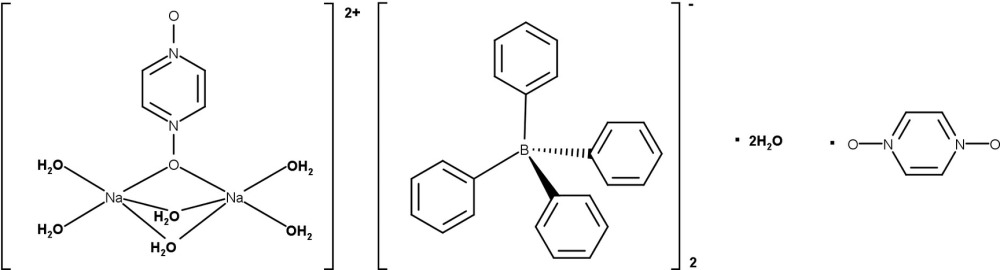


We have previously reported the structures of several three-dimensional coordination networks of the type {[*Ln*(pzdo)_4_](ClO_4_)_3_}_*n*_, with *Ln* = Nd (Quinn-Elmore *et al.*, 2010*a* ▸), Dy (Quinn-Elmore *et al.*, 2010*b* ▸), Ho (Buchner *et al.*, 2010*a* ▸), and Er (Buchner *et al.*, 2010*b* ▸), which all are isostructural to the previously reported La, Ce, Pr, Sm, Eu, Gd, Tb and Y coordination networks (Sun *et al.*, 2004 ▸). In an attempt to synthesize a novel lanthanide coordination polymer with pzdo ligands and tetra­phenyl­borate (BPh_4_^−^) anions, crystals of the title compound, [{Na(H_2_O)_2_}_2_(μ-H_2_O)_2_(μ-pzdo)][B(C_6_H_5_)_4_]_2_·2H_2_O·pzdo, were isolated instead.

## Structural commentary   

**Figure 1:**
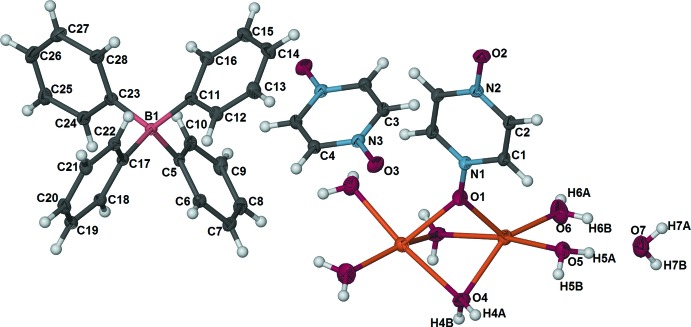
The mol­ecular entities in the crystal structure of [{Na(H_2_O)_2_}_2_(μ-H_2_O)_2_(μ-pzdo)][B(Ph)_4_]_2_·2H_2_O·pzdo drawn with displacement ellipsoids at the 50% probability level. Labeled atoms are related to unlabeled atoms by the symmetry operations: −*x* + 1, *y*, −*z* + 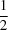 for [{Na(H_2_O)_2_}_2_(μ-H_2_O)_2_(μ-pzdo)]^2+^ and by −*x* + 1, −*y* + 1, −*z* for the solvent pzdo mol­ecule (C3, C4, N3, and O3). Only those hydrogen atoms whose positions were refined are labeled.

The asymmetric unit of the title compound contains one Na^I^ atom, half of a coordinating pzdo ligand, two terminal water ligands, one bridging water ligand, one tetra­phenyl­borate anion, half of a solvent pzdo mol­ecule and one solvent water mol­ecule (Fig. 1 ▸). The Na^I^ atom displays a distorted square-pyramidal coordination sphere defined by two O atoms of terminal water ligands, two O atoms of bridging water ligands and one O atom of the bridging pzdo ligand. The bridging water and pzdo ligands link two Na^I^ atoms to form a dinuclear cation, [{Na(H_2_O)_2_}_2_(μ-H_2_O)_2_(μ-pzdo)]^2+^, that is located about a twofold rotation axis. The oxygen and nitro­gen atoms of the coordinating pzdo ligand (O1, O2, N1, and N2) lie on a twofold rotation axis, and the solvent pzdo mol­ecule (C3, C4, N3 O3) is located around an inversion center. The pzdo ligand bridges the Na^I^ atoms in the less commonly seen end-on fashion, while the oxygen atom (O2) of the solvent pzdo mol­ecule is involved in O—H⋯O hydrogen-bonding inter­actions with another [{Na(H_2_O)_2_}_2_(μ-H_2_O)_2_(μ-pzdo)]^2+^ cation.

## Supra­molecular features   

**Figure 2:**
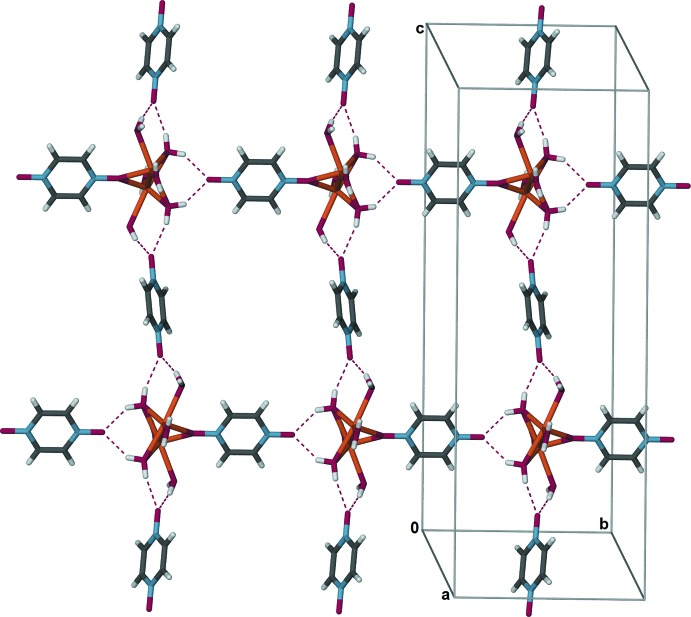
Diagram showing hydrogen-bonded [{Na(H_2_O)_2_}_2_(μ-H_2_O)_2_(μ-pzdo)]^2+^ and pzdo moieties which generate a rectangular grid parallel to the *bc* plane. Dashed lines represent O—H⋯O inter­actions between coord­inating water mol­ecules and the solvent pzdo mol­ecule (O4—H4*A*⋯O3 and O5—H5*B*⋯O3^i^) and between a coordinating water and the coordinating pzdo ligand (O4—H4*B*⋯O2^iii^). [Symmetry codes: (i) −*x* + 1, *y*, −*z* + 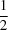; (iii) *x*, *y* − 1, *z*; (iv) −*x* + 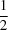, *y* + 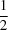, −*z* + 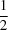.]

**Figure 3:**
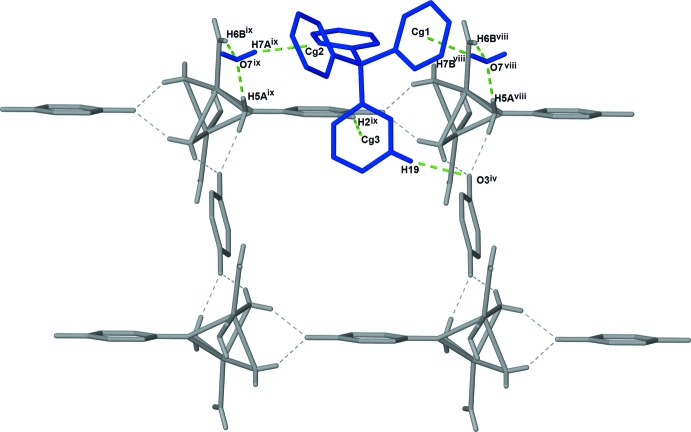
Diagram showing inter­actions linking the anion and solvent water mol­ecule to the layers. A small portion of a layer is shown with all [{Na(H_2_O)_2_}_2_(μ-H_2_O)_2_(μ-pzdo)]^2+^ and pzdo moieties represented in gray, and the hydrogen-bonding inter­actions within the layer indicated by dashed gray lines. Two solvent water mol­ecules and one anion are shown in blue. The C—H⋯O, O—H⋯O, C—H⋯π, and O—H⋯π inter­actions linking the solvent water mol­ecules and anion to the hydrogen-bonded layers are shown as dashed green lines. [Symmetry codes: (iv) −*x* + 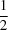, *y* + 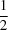, −*z* + 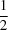; (viii) *x* − 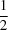, *y* + 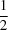, *z*; (ix) *x* − 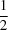, *y* − 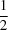, *z*.]

**Figure 4:**
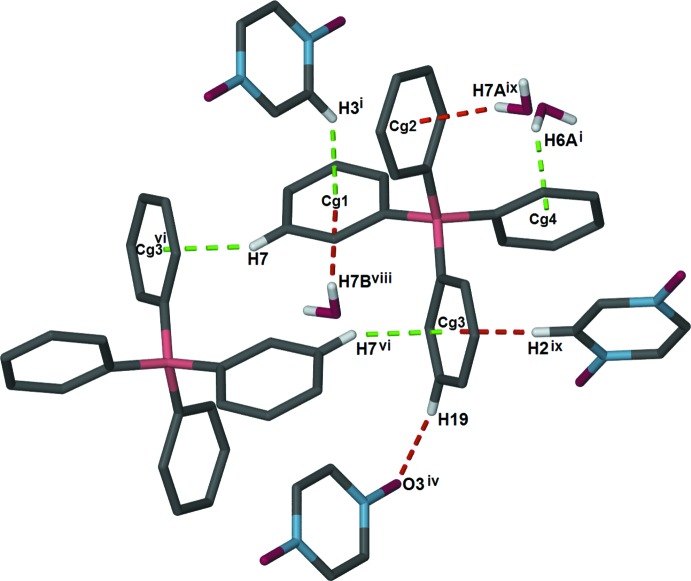
Diagram showing all C—H⋯O, O—H⋯O, C—H⋯π, and O—H⋯π inter­actions that the BPh_4_^−^ anion participates in. The C—H⋯O, C—H⋯π and O—H⋯π inter­actions responsible for linking the anion to a layer are shown as dashed red lines. The C—H⋯π and O—H⋯π inter­actions responsible for linking the layers into a three-dimensional framework are shown as dashed green lines. [Symmetry codes: (i) −*x* + 1, *y*, −*z* + 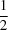; (iv) −*x* + 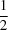, *y* + 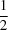, −*z* + 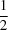; (vi) −*x* + 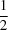, −*y* + 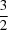, −*z* + 1; (viii) *x* − 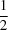, *y* + 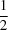, *z*; (ix) *x* − 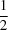, *y* − 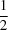, *z*.]

Three unique C—H⋯O hydrogen-bonding inter­actions between the [{Na(H_2_O)_2_}_2_(μ-H_2_O)_2_(μ-pzdo)]^2+^ cations and pzdo solvent moieties generate rectangular grid-like layers parallel to the *bc* plane. These inter­actions involve the bridging water ligand and the solvent pzdo mol­ecule (O4—H4*A*⋯O3), a terminal water ligand and the solvent pzdo mol­ecule (O5—H5*B*⋯O3^i^), and the bridging water ligand and the coordinating pzdo ligand (O4—H4*B*⋯ O2^iii^) (see Table 1 ▸ for symmetry codes; Fig. 2 ▸). Additional inter­actions link the anion and solvent water mol­ecule to the layer (Fig. 3 ▸.). The anion is linked through C—H⋯O and C—H⋯π inter­actions with the solvent pzdo mol­ecule (C19—H19⋯O3^iv^ and C2—H2⋯*Cg*3^v^). The solvent water mol­ecule accepts two hydrogen bonds from coordinating water mol­ecules (O5—H5*A*⋯O7 and O6—H6*B*⋯O7) and inter­acts with two anions through O—H⋯π inter­actions (O7—H7*A*⋯*Cg*2^v^ and O7—H7*B*⋯ *Cg*1^vii^). While all of the aforementioned inter­actions occur within a layer, additional C—H⋯π and O—H⋯π inter­actions with the tetra­phenyl­borate anions (C3—H3⋯*Cg*1^i^, O6—H6*A*⋯*Cg*4^i^, and C7—H7⋯*Cg*3^vi^) link the layers into a complex three-dimensional network (Table 1 ▸, Fig. 4 ▸).

## Database survey   

A survey of the Cambridge Structural Database (CSD, Version 5.36, November 2014; Groom & Allen, 2014 ▸) returned hits for 37 structures with pyrazine *N,N*’-dioxide. Three structures are reported for the pzdo mol­ecule. Five structures are reported for pzdo as part of a co-crystal. Fourteen structures are reported where pzdo coordinates to a transition metal and acts as a bridging ligand in a coordination network. Twelve structures are reported where pzdo coordinates to a lanthanide metal and acts as a bridging ligand in a coordination network. In all 26 reported coordination networks, pzdo bridges metal atoms in an end-to-end fashion. Two structures for mixed metal (Na^I^/Tb^III^ and Na^I^/Er^III^) coordination networks with *p*-sulfonato­calix[4]arene are reported where the Na^I^ cation is coordinated by a terminal pzdo ligand, and the structure of the mixed metal coordination network (Na^I^/La^III^) with sulfonato­calix[4]arene is reported where pzdo is included in the structure as a clathrate (Zheng *et al.*, 2008 ▸). One final structure of note deposited after the November 2014 release of the CSD is that of a mixed metal (Na^I^/W^V^) coordination network where pzdo bridges Na^I^ atoms in both end-to-end and end-on modes (Podgajny *et al.*, 2014 ▸).

## Synthesis and crystallization   

Pyrazine-*N,N*′-dioxide was synthesized from pyrazine according to the method of Simpson *et al.* (1963 ▸). All other chemicals were obtained from commercial sources and used without further purification. Initially, NaBPh_4_ (0.0821 g, 0.240 mmol), pzdo (0.0171 g, 0.152 mmol) and 40%_wt_ aqueous Ho(ClO_4_)_3_ (14.8 µl, 0.0201 mmol), were combined in 25 ml of methanol to form a cloudy solution, and colorless crystals of the title compound were obtained upon slow evaporation of the solvent. Further studies showed that crystals of the title compound can also be isolated in the absence of the lanthanide salt. In this case, NaBPh_4_ (0.0257 g, 0.0750 mmol) and pzdo (0.0171 g, 0.152 mmol) were combined in 12.5 ml methanol and 1.1 ml of water to form a cloudy solution which yielded colorless crystals of the title compound upon slow evaporation of the solvent.

## Refinement   

Crystal data, data collection and structure refinement details are summarized in Table 2 ▸. All aromatic H atoms were positioned geometrically and refined using a riding model with C—H = 0.95 Å and with *U*_iso_(H) = 1.2 times *U*_eq_(C). The positions of water H atoms were located from difference Fourier maps and the O—H distances in the water mol­ecules were restrained to 0.85 (2) Å. *U*_iso_ parameters of water H atoms were refined freely.

## Supplementary Material

Crystal structure: contains datablock(s) I. DOI: 10.1107/S205698901502071X/wm5232sup1.cif

Structure factors: contains datablock(s) I. DOI: 10.1107/S205698901502071X/wm5232Isup2.hkl

CCDC reference: 1434594

Additional supporting information:  crystallographic information; 3D view; checkCIF report

## Figures and Tables

**Table 1:** Hydrogen-bond geometry (Å, °) *Cg*1, *Cg*2, *Cg*3 and *Cg*4 are the centroids of the C5–C10, C11–C16, C17–C22 and C23–C28 rings, respectively.

*D*—H⋯*A*	*D*—H	H⋯*A*	*D*⋯*A*	*D*—H⋯*A*
O4—H4*A*⋯O3	0.86 (2)	2.09 (2)	2.8855 (13)	153 (2)
O4—H4*B*⋯O2^iii^	0.85 (2)	1.95 (2)	2.6948 (14)	144 (2)
O5—H5*B*⋯O3^i^	0.84 (2)	1.95 (2)	2.7655 (14)	163 (2)
O5—H5*A*⋯O7	0.86 (2)	2.00 (2)	2.8329 (16)	163 (2)
O6—H6*B*⋯O7	0.88 (2)	2.06 (2)	2.9055 (19)	160 (3)
C19—H19⋯O3^iv^	0.95	2.55	3.4884 (16)	168
C2—H2⋯*Cg*3^v^	0.95	2.40	3.2435 (14)	148
C3—H3⋯*Cg*1^i^	0.95	2.46	3.2788 (14)	144
O6—H6*A*⋯*Cg*4^i^	0.85 (3)	2.45 (3)	3.1713 (14)	144 (2)
C7—H7⋯*Cg*3^vi^	0.95	2.66	3.5365 (14)	153
O7—H7*A*⋯*Cg*2^v^	0.86 (2)	2.55 (2)	3.3871 (15)	165 (2)
O7—H7*B*⋯*Cg*1^vii^	0.85 (3)	2.59 (2)	3.4337 (15)	171 (3)

Symmetry codes: (i) 
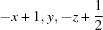
; (iii) 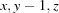; (iv) 
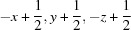
; (v) 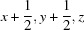; (vi) 
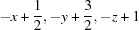
; (vii) 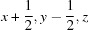.

**Table 2:** Experimental details

Crystal data	
Chemical formula	[Na_2_(C_4_H_4_N_2_O_2_)(H_2_O)_6_](BC_24_H_20_)_2_·C_4_H_4_N_2_O_2_·2H_2_O
*M*_r_	1052.71
Crystal system, space group	Monoclinic, *C*2/*c*
Temperature (K)	99
*a*, *b*, *c* (Å)	20.4224 (9), 10.1950 (4), 27.2349 (11)
β (°)	102.947 (1)
*V* (Å^3^)	5526.3 (4)
*Z*	4
Radiation type	Mo *K*α
μ (mm^−1^)	0.10
Crystal size (mm)	0.50 × 0.40 × 0.25

Data collection	
Diffractometer	Bruker SMART APEX CCD diffractometer
Absorption correction	Multi-scan (*SADABS*; Bruker, 2001 ▸)
*T*_min_, *T*_max_	0.894, 1.000
No. of measured, independent and observed [*I* > 2σ(*I*)] reflections	32437, 8464, 6996
*R*_int_	0.037
(sin θ/λ)_max_ (Å^−1^)	0.715

Refinement	
*R*[*F* ^2^ > 2σ(*F* ^2^)], *wR*(*F* ^2^), *S*	0.052, 0.140, 1.04
No. of reflections	8464
No. of parameters	377
No. of restraints	8
H-atom treatment	H atoms treated by a mixture of independent and constrained refinement
Δρ_max_, Δρ_min_ (e Å^−3^)	0.48, −0.21

Computer programs: *SMART* and *SAINT* (Bruker, 2007 ▸), *SHELXS97* and *SHELXL97* (Sheldrick, 2008 ▸), and *X-SEED* (Barbour, 2001 ▸).

## supplementary crystallographic information

### Crystal data

**Table:**

[Na_2_(C_4_H_4_N_2_O_2_)(H_2_O)_6_](BC_24_H_20_)_2_·C_4_H_4_N_2_O_2_·2H_2_O	*F*(000) = 2224
*M**_r_* = 1052.71	*D*_x_ = 1.265 Mg m^−^^3^
Monoclinic, *C*2/*c*	Mo *K*α radiation, λ = 0.71073 Å
Hall symbol: -C 2yc	Cell parameters from 14931 reflections
*a* = 20.4224 (9) Å	θ = 2.2–30.5°
*b* = 10.1950 (4) Å	µ = 0.10 mm^−^^1^
*c* = 27.2349 (11) Å	*T* = 99 K
β = 102.947 (1)°	Block, colorless
*V* = 5526.3 (4) Å^3^	0.50 × 0.40 × 0.25 mm
*Z* = 4	

### Data collection

**Table:**

Bruker SMART APEX CCD diffractometer	8464 independent reflections
Radiation source: fine-focus sealed tube	6996 reflections with *I* > 2σ(*I*)
Graphite monochromator	*R*_int_ = 0.037
ω scans	θ_max_ = 30.5°, θ_min_ = 1.5°
Absorption correction: multi-scan (*SADABS*; Bruker, 2001)	*h* = −29→29
*T*_min_ = 0.894, *T*_max_ = 1.000	*k* = −14→14
32437 measured reflections	*l* = −37→38

### Refinement

**Table:**

Refinement on *F*^2^	Primary atom site location: structure-invariant direct methods
Least-squares matrix: full	Secondary atom site location: difference Fourier map
*R*[*F*^2^ > 2σ(*F*^2^)] = 0.052	Hydrogen site location: inferred from neighbouring sites
*wR*(*F*^2^) = 0.140	H atoms treated by a mixture of independent and constrained refinement
*S* = 1.04	*w* = 1/[σ^2^(*F*_o_^2^) + (0.0739*P*)^2^ + 3.1014*P*] where *P* = (*F*_o_^2^ + 2*F*_c_^2^)/3
8464 reflections	(Δ/σ)_max_ = 0.001
377 parameters	Δρ_max_ = 0.48 e Å^−^^3^
8 restraints	Δρ_min_ = −0.21 e Å^−^^3^

### Special details

**Table:**

Geometry. All e.s.d.'s (except the e.s.d. in the dihedral angle between two l.s. planes) are estimated using the full covariance matrix. The cell e.s.d.'s are taken into account individually in the estimation of e.s.d.'s in distances, angles and torsion angles; correlations between e.s.d.'s in cell parameters are only used when they are defined by crystal symmetry. An approximate (isotropic) treatment of cell e.s.d.'s is used for estimating e.s.d.'s involving l.s. planes.
Refinement. Refinement of *F*^2^ against ALL reflections. The weighted *R*-factor *wR* and goodness of fit *S* are based on *F*^2^, conventional *R*-factors *R* are based on *F*, with *F* set to zero for negative *F*^2^. The threshold expression of *F*^2^ > σ(*F*^2^) is used only for calculating *R*-factors(gt) *etc*. and is not relevant to the choice of reflections for refinement. *R*-factors based on *F*^2^ are statistically about twice as large as those based on *F*, and *R*- factors based on ALL data will be even larger.

### Fractional atomic coordinates and isotropic or equivalent isotropic displacement parameters (Å^2^)

**Table:**

	*x*	*y*	*z*	*U*_iso_*/*U*_eq_	
Na1	0.57681 (3)	0.53904 (5)	0.27762 (2)	0.02408 (12)	
O1	0.5000	0.70101 (12)	0.2500	0.0302 (3)	
O2	0.5000	1.22491 (12)	0.2500	0.0253 (3)	
O3	0.48519 (5)	0.52324 (10)	0.09489 (3)	0.0272 (2)	
O4	0.52081 (5)	0.44163 (9)	0.19893 (4)	0.0266 (2)	
O5	0.61154 (5)	0.62448 (11)	0.35909 (4)	0.0316 (2)	
O6	0.68618 (6)	0.53238 (14)	0.27608 (5)	0.0441 (3)	
O7	0.75011 (6)	0.56103 (14)	0.38213 (5)	0.0434 (3)	
N1	0.5000	0.82883 (14)	0.2500	0.0210 (3)	
N2	0.5000	1.09705 (14)	0.2500	0.0195 (3)	
N3	0.49248 (5)	0.51216 (10)	0.04872 (4)	0.0196 (2)	
C1	0.53467 (6)	0.89566 (12)	0.29054 (5)	0.0214 (2)	
H1	0.5591	0.8492	0.3191	0.026*	
C2	0.53468 (6)	1.02966 (12)	0.29060 (5)	0.0209 (2)	
H2	0.5591	1.0760	0.3192	0.025*	
C3	0.54999 (6)	0.55124 (12)	0.03612 (5)	0.0215 (2)	
H3	0.5855	0.5872	0.0612	0.026*	
C4	0.44262 (6)	0.46070 (12)	0.01235 (5)	0.0212 (2)	
H4	0.4021	0.4326	0.0207	0.025*	
C5	0.28988 (5)	0.68369 (11)	0.39692 (4)	0.0165 (2)	
C6	0.29439 (6)	0.74729 (12)	0.44320 (4)	0.0204 (2)	
H6	0.2623	0.7264	0.4624	0.024*	
C7	0.34389 (6)	0.83982 (13)	0.46232 (5)	0.0240 (2)	
H7	0.3450	0.8808	0.4938	0.029*	
C8	0.39154 (6)	0.87171 (13)	0.43493 (5)	0.0257 (3)	
H8	0.4258	0.9337	0.4478	0.031*	
C9	0.38860 (6)	0.81212 (13)	0.38855 (5)	0.0249 (3)	
H9	0.4208	0.8336	0.3695	0.030*	
C10	0.33829 (6)	0.72066 (12)	0.37003 (4)	0.0202 (2)	
H10	0.3367	0.6819	0.3381	0.024*	
C11	0.28603 (6)	0.43432 (11)	0.40579 (4)	0.0176 (2)	
C12	0.28816 (6)	0.39219 (12)	0.45536 (4)	0.0203 (2)	
H12	0.2577	0.4301	0.4731	0.024*	
C13	0.33308 (6)	0.29710 (13)	0.47952 (5)	0.0244 (2)	
H13	0.3325	0.2711	0.5129	0.029*	
C14	0.37864 (7)	0.24013 (13)	0.45489 (5)	0.0281 (3)	
H14	0.4094	0.1754	0.4712	0.034*	
C15	0.37846 (6)	0.27962 (13)	0.40590 (5)	0.0264 (3)	
H15	0.4095	0.2421	0.3886	0.032*	
C16	0.33281 (6)	0.37423 (12)	0.38211 (5)	0.0217 (2)	
H16	0.3333	0.3990	0.3486	0.026*	
C17	0.16906 (6)	0.57084 (11)	0.39863 (4)	0.0164 (2)	
C18	0.13473 (6)	0.69104 (11)	0.39796 (4)	0.0186 (2)	
H18	0.1562	0.7692	0.3909	0.022*	
C19	0.07050 (6)	0.69938 (12)	0.40727 (4)	0.0215 (2)	
H19	0.0487	0.7820	0.4058	0.026*	
C20	0.03822 (6)	0.58705 (13)	0.41870 (5)	0.0232 (2)	
H20	−0.0052	0.5927	0.4257	0.028*	
C21	0.07032 (6)	0.46643 (12)	0.41976 (5)	0.0211 (2)	
H21	0.0488	0.3889	0.4275	0.025*	
C22	0.13431 (6)	0.45933 (11)	0.40948 (4)	0.0183 (2)	
H22	0.1551	0.3759	0.4098	0.022*	
C23	0.21392 (6)	0.54690 (11)	0.31871 (4)	0.0181 (2)	
C24	0.19493 (6)	0.66032 (12)	0.28925 (4)	0.0209 (2)	
H24	0.2043	0.7439	0.3047	0.025*	
C25	0.16291 (6)	0.65426 (14)	0.23831 (5)	0.0254 (3)	
H25	0.1507	0.7329	0.2198	0.030*	
C26	0.14892 (7)	0.53359 (14)	0.21466 (5)	0.0273 (3)	
H26	0.1270	0.5290	0.1800	0.033*	
C27	0.16730 (7)	0.41963 (14)	0.24223 (5)	0.0268 (3)	
H27	0.1583	0.3365	0.2264	0.032*	
C28	0.19901 (6)	0.42686 (12)	0.29327 (5)	0.0220 (2)	
H28	0.2109	0.3477	0.3115	0.026*	
B1	0.23971 (6)	0.55838 (12)	0.37996 (5)	0.0161 (2)	
H4A	0.5221 (12)	0.451 (2)	0.1677 (6)	0.057 (7)*	
H5A	0.6528 (8)	0.605 (2)	0.3724 (8)	0.053 (6)*	
H6A	0.7091 (14)	0.514 (3)	0.2547 (9)	0.090 (9)*	
H5B	0.5879 (10)	0.597 (2)	0.3788 (8)	0.059 (7)*	
H4B	0.5158 (11)	0.3594 (16)	0.2028 (8)	0.059 (6)*	
H7A	0.7775 (12)	0.625 (2)	0.3922 (10)	0.086 (9)*	
H6B	0.7144 (13)	0.543 (3)	0.3054 (8)	0.082 (9)*	
H7B	0.7749 (15)	0.496 (2)	0.3939 (12)	0.101 (11)*	

### Atomic displacement parameters (Å^2^)

**Table:**

	*U*^11^	*U*^22^	*U*^33^	*U*^12^	*U*^13^	*U*^23^
Na1	0.0223 (2)	0.0254 (3)	0.0240 (3)	0.00197 (19)	0.00423 (19)	0.00052 (19)
O1	0.0338 (7)	0.0136 (6)	0.0365 (7)	0.000	−0.0068 (6)	0.000
O2	0.0339 (7)	0.0137 (5)	0.0280 (6)	0.000	0.0063 (5)	0.000
O3	0.0331 (5)	0.0334 (5)	0.0162 (4)	0.0025 (4)	0.0080 (4)	−0.0001 (3)
O4	0.0377 (5)	0.0205 (4)	0.0220 (4)	0.0011 (4)	0.0075 (4)	0.0000 (3)
O5	0.0267 (5)	0.0407 (6)	0.0266 (5)	−0.0035 (4)	0.0046 (4)	0.0008 (4)
O6	0.0254 (5)	0.0644 (8)	0.0446 (7)	−0.0014 (5)	0.0124 (5)	−0.0088 (6)
O7	0.0276 (6)	0.0469 (7)	0.0502 (7)	0.0008 (5)	−0.0030 (5)	0.0040 (6)
N1	0.0220 (7)	0.0159 (6)	0.0225 (7)	0.000	−0.0004 (5)	0.000
N2	0.0217 (7)	0.0156 (6)	0.0211 (7)	0.000	0.0046 (5)	0.000
N3	0.0221 (5)	0.0196 (5)	0.0168 (4)	0.0010 (4)	0.0037 (4)	0.0005 (3)
C1	0.0214 (5)	0.0211 (6)	0.0193 (5)	−0.0001 (4)	−0.0009 (4)	0.0006 (4)
C2	0.0221 (5)	0.0205 (5)	0.0183 (5)	−0.0013 (4)	0.0009 (4)	−0.0007 (4)
C3	0.0206 (5)	0.0217 (5)	0.0203 (5)	−0.0036 (4)	0.0003 (4)	0.0004 (4)
C4	0.0180 (5)	0.0238 (6)	0.0212 (5)	−0.0020 (4)	0.0032 (4)	0.0021 (4)
C5	0.0155 (5)	0.0173 (5)	0.0158 (5)	0.0009 (4)	0.0012 (4)	0.0021 (4)
C6	0.0200 (5)	0.0217 (5)	0.0192 (5)	−0.0012 (4)	0.0037 (4)	−0.0014 (4)
C7	0.0254 (6)	0.0225 (6)	0.0216 (6)	−0.0020 (4)	0.0001 (4)	−0.0035 (4)
C8	0.0233 (6)	0.0222 (6)	0.0283 (6)	−0.0057 (5)	−0.0011 (5)	0.0019 (5)
C9	0.0207 (5)	0.0278 (6)	0.0256 (6)	−0.0041 (5)	0.0039 (4)	0.0069 (5)
C10	0.0205 (5)	0.0229 (6)	0.0167 (5)	−0.0013 (4)	0.0028 (4)	0.0026 (4)
C11	0.0165 (5)	0.0165 (5)	0.0193 (5)	−0.0002 (4)	0.0031 (4)	0.0009 (4)
C12	0.0192 (5)	0.0210 (5)	0.0209 (5)	0.0006 (4)	0.0052 (4)	0.0025 (4)
C13	0.0256 (6)	0.0237 (6)	0.0231 (6)	0.0010 (5)	0.0035 (5)	0.0066 (4)
C14	0.0271 (6)	0.0226 (6)	0.0328 (7)	0.0076 (5)	0.0030 (5)	0.0053 (5)
C15	0.0246 (6)	0.0253 (6)	0.0293 (6)	0.0074 (5)	0.0064 (5)	−0.0008 (5)
C16	0.0217 (5)	0.0219 (6)	0.0219 (5)	0.0028 (4)	0.0058 (4)	0.0005 (4)
C17	0.0165 (5)	0.0184 (5)	0.0139 (5)	0.0009 (4)	0.0025 (4)	−0.0006 (4)
C18	0.0193 (5)	0.0177 (5)	0.0179 (5)	0.0001 (4)	0.0021 (4)	−0.0011 (4)
C19	0.0202 (5)	0.0224 (6)	0.0210 (5)	0.0050 (4)	0.0028 (4)	−0.0024 (4)
C20	0.0158 (5)	0.0311 (6)	0.0231 (6)	0.0021 (4)	0.0050 (4)	0.0006 (5)
C21	0.0179 (5)	0.0240 (6)	0.0213 (5)	−0.0022 (4)	0.0037 (4)	0.0034 (4)
C22	0.0180 (5)	0.0188 (5)	0.0176 (5)	0.0002 (4)	0.0026 (4)	0.0008 (4)
C23	0.0178 (5)	0.0205 (5)	0.0168 (5)	−0.0001 (4)	0.0055 (4)	−0.0008 (4)
C24	0.0212 (5)	0.0228 (6)	0.0182 (5)	0.0005 (4)	0.0036 (4)	0.0001 (4)
C25	0.0235 (6)	0.0321 (7)	0.0198 (6)	0.0011 (5)	0.0032 (4)	0.0035 (5)
C26	0.0246 (6)	0.0402 (8)	0.0165 (5)	−0.0029 (5)	0.0032 (4)	−0.0028 (5)
C27	0.0280 (6)	0.0308 (7)	0.0218 (6)	−0.0050 (5)	0.0063 (5)	−0.0079 (5)
C28	0.0228 (6)	0.0238 (6)	0.0198 (5)	−0.0004 (4)	0.0054 (4)	−0.0023 (4)
B1	0.0162 (5)	0.0166 (5)	0.0152 (5)	0.0000 (4)	0.0033 (4)	−0.0004 (4)

### Geometric parameters (Å, º)

**Table:**

Na1—O6	2.2444 (13)	C9—C10	1.3954 (17)
Na1—O1	2.2857 (10)	C9—H9	0.9500
Na1—O5	2.3410 (12)	C10—H10	0.9500
Na1—O4	2.4059 (11)	C11—C16	1.4070 (16)
Na1—O4^i^	2.4371 (12)	C11—C12	1.4083 (16)
O1—N1	1.3031 (19)	C11—B1	1.6404 (17)
O1—Na1^i^	2.2857 (10)	C12—C13	1.3942 (17)
O2—N2	1.3035 (18)	C12—H12	0.9500
O3—N3	1.3040 (13)	C13—C14	1.3903 (19)
O4—Na1^i^	2.4371 (12)	C13—H13	0.9500
O4—H4A	0.862 (16)	C14—C15	1.3927 (19)
O4—H4B	0.854 (16)	C14—H14	0.9500
O5—H5A	0.863 (15)	C15—C16	1.3959 (17)
O5—H5B	0.844 (16)	C15—H15	0.9500
O6—H6A	0.844 (17)	C16—H16	0.9500
O6—H6B	0.880 (17)	C17—C22	1.4062 (16)
O7—H7A	0.861 (17)	C17—C18	1.4100 (16)
O7—H7B	0.855 (18)	C17—B1	1.6386 (17)
N1—C1^i^	1.3544 (14)	C18—C19	1.3932 (16)
N1—C1	1.3544 (14)	C18—H18	0.9500
N2—C2^i^	1.3583 (14)	C19—C20	1.3910 (18)
N2—C2	1.3584 (14)	C19—H19	0.9500
N3—C3	1.3552 (16)	C20—C21	1.3909 (18)
N3—C4	1.3570 (15)	C20—H20	0.9500
C1—C2	1.3661 (17)	C21—C22	1.3982 (16)
C1—H1	0.9500	C21—H21	0.9500
C2—H2	0.9500	C22—H22	0.9500
C3—C4^ii^	1.3673 (17)	C23—C28	1.4056 (17)
C3—H3	0.9500	C23—C24	1.4113 (16)
C4—C3^ii^	1.3672 (17)	C23—B1	1.6373 (17)
C4—H4	0.9500	C24—C25	1.3962 (16)
C5—C6	1.4020 (16)	C24—H24	0.9500
C5—C10	1.4073 (16)	C25—C26	1.3884 (19)
C5—B1	1.6381 (17)	C25—H25	0.9500
C6—C7	1.3953 (17)	C26—C27	1.389 (2)
C6—H6	0.9500	C26—H26	0.9500
C7—C8	1.3908 (19)	C27—C28	1.3978 (17)
C7—H7	0.9500	C27—H27	0.9500
C8—C9	1.3909 (19)	C28—H28	0.9500
C8—H8	0.9500		
			
O6—Na1—O1	128.92 (5)	C9—C10—H10	118.8
O6—Na1—O5	86.41 (5)	C5—C10—H10	118.8
O1—Na1—O5	94.76 (4)	C16—C11—C12	115.36 (10)
O6—Na1—O4	104.32 (5)	C16—C11—B1	121.65 (10)
O1—Na1—O4	81.42 (3)	C12—C11—B1	122.49 (10)
O5—Na1—O4	168.66 (4)	C13—C12—C11	122.70 (11)
O6—Na1—O4^i^	150.29 (5)	C13—C12—H12	118.7
O1—Na1—O4^i^	80.75 (3)	C11—C12—H12	118.7
O5—Na1—O4^i^	89.72 (4)	C14—C13—C12	120.24 (12)
O4—Na1—O4^i^	79.15 (4)	C14—C13—H13	119.9
N1—O1—Na1^i^	136.26 (3)	C12—C13—H13	119.9
N1—O1—Na1	136.26 (3)	C13—C14—C15	118.88 (12)
Na1^i^—O1—Na1	87.49 (5)	C13—C14—H14	120.6
Na1—O4—Na1^i^	81.48 (4)	C15—C14—H14	120.6
Na1—O4—H4A	136.1 (15)	C14—C15—C16	120.17 (12)
Na1^i^—O4—H4A	115.0 (15)	C14—C15—H15	119.9
Na1—O4—H4B	109.9 (15)	C16—C15—H15	119.9
Na1^i^—O4—H4B	103.9 (15)	C15—C16—C11	122.66 (11)
H4A—O4—H4B	105 (2)	C15—C16—H16	118.7
Na1—O5—H5A	111.9 (15)	C11—C16—H16	118.7
Na1—O5—H5B	112.6 (16)	C22—C17—C18	115.62 (10)
H5A—O5—H5B	108 (2)	C22—C17—B1	121.57 (10)
Na1—O6—H6A	137 (2)	C18—C17—B1	122.22 (10)
Na1—O6—H6B	115.5 (19)	C19—C18—C17	122.44 (11)
H6A—O6—H6B	108 (3)	C19—C18—H18	118.8
H7A—O7—H7B	100 (3)	C17—C18—H18	118.8
O1—N1—C1^i^	120.20 (7)	C20—C19—C18	120.22 (11)
O1—N1—C1	120.20 (7)	C20—C19—H19	119.9
C1^i^—N1—C1	119.59 (15)	C18—C19—H19	119.9
O2—N2—C2^i^	120.38 (7)	C21—C20—C19	119.19 (11)
O2—N2—C2	120.38 (7)	C21—C20—H20	120.4
C2^i^—N2—C2	119.24 (14)	C19—C20—H20	120.4
O3—N3—C3	120.75 (10)	C20—C21—C22	119.93 (11)
O3—N3—C4	120.55 (10)	C20—C21—H21	120.0
C3—N3—C4	118.69 (10)	C22—C21—H21	120.0
N1—C1—C2	120.25 (11)	C21—C22—C17	122.59 (11)
N1—C1—H1	119.9	C21—C22—H22	118.7
C2—C1—H1	119.9	C17—C22—H22	118.7
N2—C2—C1	120.33 (11)	C28—C23—C24	115.57 (11)
N2—C2—H2	119.8	C28—C23—B1	123.30 (10)
C1—C2—H2	119.8	C24—C23—B1	120.36 (10)
N3—C3—C4^ii^	120.51 (11)	C25—C24—C23	122.44 (12)
N3—C3—H3	119.7	C25—C24—H24	118.8
C4^ii^—C3—H3	119.7	C23—C24—H24	118.8
N3—C4—C3^ii^	120.80 (11)	C26—C25—C24	120.14 (12)
N3—C4—H4	119.6	C26—C25—H25	119.9
C3^ii^—C4—H4	119.6	C24—C25—H25	119.9
C6—C5—C10	115.58 (10)	C25—C26—C27	119.20 (12)
C6—C5—B1	121.67 (10)	C25—C26—H26	120.4
C10—C5—B1	122.01 (10)	C27—C26—H26	120.4
C7—C6—C5	122.97 (11)	C26—C27—C28	120.17 (12)
C7—C6—H6	118.5	C26—C27—H27	119.9
C5—C6—H6	118.5	C28—C27—H27	119.9
C8—C7—C6	119.57 (12)	C27—C28—C23	122.48 (12)
C8—C7—H7	120.2	C27—C28—H28	118.8
C6—C7—H7	120.2	C23—C28—H28	118.8
C9—C8—C7	119.46 (11)	C23—B1—C5	112.40 (9)
C9—C8—H8	120.3	C23—B1—C17	102.54 (9)
C7—C8—H8	120.3	C5—B1—C17	113.00 (9)
C8—C9—C10	119.92 (12)	C23—B1—C11	113.87 (9)
C8—C9—H9	120.0	C5—B1—C11	102.49 (9)
C10—C9—H9	120.0	C17—B1—C11	112.96 (9)
C9—C10—C5	122.48 (11)		

Symmetry codes: (i) −*x*+1, *y*, −*z*+1/2; (ii) −*x*+1, −*y*+1, −*z*.

### Hydrogen-bond geometry (Å, º)

*Cg*1, *Cg*2, *Cg*3 and *Cg*4 are the centroids of the C5–C10, C11–C16, C17–C22 and C23–C28 rings, respectively.

**Table:**

*D*—H···*A*	*D*—H	H···*A*	*D*···*A*	*D*—H···*A*
O4—H4*A*···O3	0.86 (2)	2.09 (2)	2.8855 (13)	153 (2)
O4—H4*B*···O2^iii^	0.85 (2)	1.95 (2)	2.6948 (14)	144 (2)
O5—H5*B*···O3^i^	0.84 (2)	1.95 (2)	2.7655 (14)	163 (2)
O5—H5*A*···O7	0.86 (2)	2.00 (2)	2.8329 (16)	163 (2)
O6—H6*B*···O7	0.88 (2)	2.06 (2)	2.9055 (19)	160 (3)
C19—H19···O3^iv^	0.95	2.55	3.4884 (16)	168
C2—H2···*Cg*3^v^	0.95	2.40	3.2435 (14)	148
C3—H3···*Cg*1^i^	0.95	2.46	3.2788 (14)	144
O6—H6*A*···*Cg*4^i^	0.85 (3)	2.45 (3)	3.1713 (14)	144 (2)
C7—H7···*Cg*3^vi^	0.95	2.66	3.5365 (14)	153
O7—H7*A*···*Cg*2^v^	0.86 (2)	2.55 (2)	3.3871 (15)	165 (2)
O7—H7*B*···*Cg*1^vii^	0.85 (3)	2.59 (2)	3.4337 (15)	171 (3)

Symmetry codes: (i) −*x*+1, *y*, −*z*+1/2; (iii) *x*, *y*−1, *z*; (iv) −*x*+1/2, *y*+1/2, −*z*+1/2; (v) *x*+1/2, *y*+1/2, *z*; (vi) −*x*+1/2, −*y*+3/2, −*z*+1; (vii) *x*+1/2, *y*−1/2, *z*.
